# An Empirical Method for Establishing Positional Confidence Intervals Tailored for Composite Interval Mapping of QTL

**DOI:** 10.1371/journal.pone.0009039

**Published:** 2010-02-09

**Authors:** Andrew Crossett, Nick Lauter, Tanzy M. Love

**Affiliations:** 1 Department of Statistics, Carnegie Mellon University, Pittsburgh, Pennsylvania, United States of America; 2 USDA-ARS Corn Insects and Crop Genetics Research Unit and Departments of Plant Pathology and Agronomy, Iowa State University, Ames, Iowa, United States of America; Virginia Tech, United States of America

## Abstract

**Background:**

Improved genetic resolution and availability of sequenced genomes have made positional cloning of moderate-effect QTL realistic in several systems, emphasizing the need for precise and accurate derivation of positional confidence intervals (CIs) for QTL. Support interval (SI) methods based on the shape of the QTL likelihood curve have proven adequate for standard interval mapping, but have not been shown to be appropriate for use with composite interval mapping (CIM), which is one of the most commonly used QTL mapping methods.

**Results:**

Based on a non-parametric confidence interval (NPCI) method designed for use with the Haley-Knott regression method for mapping QTL, a CIM-specific method (CIM-NPCI) was developed to appropriately account for the selection of background markers during analysis of bootstrap-resampled data sets. Coverage probabilities and interval widths resulting from use of the NPCI, SI, and CIM-NPCI methods were compared in a series of simulations analyzed via CIM, wherein four genetic effects were simulated in chromosomal regions with distinct marker densities while heritability was fixed at 0.6 for a population of 200 isolines. CIM-NPCIs consistently capture the simulated QTL across these conditions while slightly narrower SIs and NPCIs fail at unacceptably high rates, especially in genomic regions where marker density is high, which is increasingly common for real studies. The effects of a known CIM bias toward locating QTL peaks at markers were also investigated for each marker density case. Evaluation of sub-simulations that varied according to the positions of simulated effects relative to the nearest markers showed that the CIM-NPCI method overcomes this bias, offering an explanation for the improved coverage probabilities when marker densities are high.

**Conclusions:**

Extensive simulation studies herein demonstrate that the QTL confidence interval methods typically used to positionally evaluate CIM results can be dramatically improved by accounting for the procedural complexity of CIM via an empirical approach, CIM-NPCI. Confidence intervals are a critical measure of QTL utility, but have received inadequate treatment due to a perception that QTL mapping is not sufficiently precise for procedural improvements to matter. Technological advances will continue to challenge this assumption, creating even more need for the current improvement to be refined.

## Introduction

Through genome-wide searches for statistical associations between genotypes and phenotypes, quantitative trait locus (QTL) analysis simultaneously locates genetic effects on the trait of interest to positions within the genome and characterizes the relative phenotypic consequences of carrying certain natural alleles at these loci [1]. Since the inception of this approach several decades ago, these alluring capabilities have driven innovations in genetic marker technologies, population design and statistical methods, largely targeted at realizing the potential of this method to clone the nucleotide polymorphisms that cause the natural phenotypic variations we observe [2].

For many QTL studies, genetic resolution remains limited by the number of recombination events and/or the marker density required to fully delineate them. The number of recombination events is a function of population type and size, and has been overcome in several systems by elegant breeding designs, particularly among plants. For example, in maize, three intermated recombinant inbred line (iRIL) populations have been created [3; A. Charcosset, unpublished; N. Lauter and S. Moose, unpublished]. These are dramatically enriched for the number of recombination events per line, such that genetic resolution is improved by up to 50-fold compared to traditional RIL populations [4]. In order to be realized, gains in genetic resolution were accompanied by commensurate improvements in genetic marker density [5], [6]. Although none of these maize populations is fully resolved by markers, achievement of appropriately high marker density will fade as a major limitation, since transcript-derived markers have been shown to be reliable for generation of thousands of new markers per experiment [7], and suitable transcript profiling platforms exist for at least 14 crops as well as for all animal model systems. More recently, experimental populations with even higher genetic resolution have been developed for public use [8], [9]. Collectively, these advances place the burden on statisticians to evolve methods that accommodate these gains in QTL resolution, such that statistical methodologies are not limiting in this process.

There has been extensive literature written on identifying QTL. Much of this literature is statistical in nature and, as with any statistical problem, it is not enough to simply state an estimate of a parameter of interest without indicating some measure of uncertainty. Especially in cases when fine-mapping is being pursued toward goals such as positional cloning or establishing pleiotropic action, a hard set of bounds that contain a QTL with a certain confidence is of great interest. Thus, the statistical challenge has turned from QTL estimation to the construction of confidence intervals for these locations.

The first and most widely used confidence estimation method is the LOD drop-off, or support interval (SI) method [10]. For an estimated QTL, a SI is determined by plotting the LOD score (obtained from a chosen QTL estimation method) along a chromosome to generate the LOD curve and then by following the curve from the peak to its prescribed drop in LOD value on each side. It has been shown in previous studies of standard interval mapping that in order for a SI to have 95% coverage, the LOD drop should be between 1.5 and 2.0 units, depending on the length of the genome and marker density [11], [12]. SI widths intimately depend on the shape of the LOD profile, specifically the steepness of the drops flanking the QTL peak.

Another approach to constructing confidence intervals for estimated QTL is to use a non-parametric bootstrap confidence interval (NPCI) method that repeatedly samples *n* observations, with replacement, from the original sample of size *n* [13], [14]. For each resampled data set, the location of the QTL of interest is estimated using a particular QTL estimation method. This process is repeated *B* times and a 95% NPCI is constructed by ordering the *B* estimated QTL peaks along the chromosome and reporting the 2.5 and 97.5 percentiles as α = 0.05 positional bounds for the QTL [14]. Alternatively, the CI can be assumed to be symmetric and calculated from the replications accordingly [13]. NPCI methods are not as dependent as SI methods on the shape of the LOD profile around the maximum LOD value [14].

Both SI and NPCI methods have previously been used to analyze QTL positions estimated by standard interval mapping (SIM) [10], [14]. SIM tests for the presence of a QTL at any location along the genome using the nearest fully informative genetic markers (flanking markers) that capture the position in question. Composite interval mapping (CIM) does this as well, but is much more widely used because it has been shown to localize QTL more precisely [15], [16], [17]. When testing a putative QTL, CIM includes additional markers as covariates in the model to help control for effects of other QTL.

**Figure 1:**
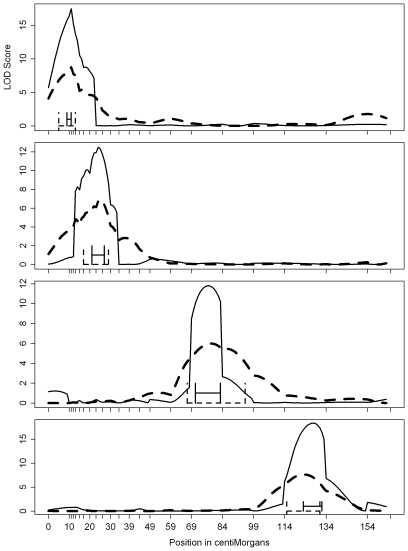
LOD profile plot for one simulated data set with *n* = 200 individuals. In the simulation there are four chromosomes where each chromosome contains a single QTL. The dashed line indicates the LOD profile when using SIM and the solid line indicates the LOD profile when using CIM. SIs where drawn under each profile. Marker locations are indicated by tick marks.

There are marked differences in the shapes of LOD profiles generated from SIM versus CIM (Figure 1). In addition to effects of the number of selected background markers and their minimum statistical significance, adjustable parameters which affect LOD curve shape include the distance between test interval sites and the size of the blockout window in which background markers are excluded from the model [4], [18]. When markers selected as covariates for CIM are linked to a test position, drastic changes in the LOD curve shape are caused by moderate adjustments to CIM parameters set by the user, rather than by actual changes in the likelihood of a QTL existing at the test site [19]. Naturally, such differences in profile shape alter positional confidence results more dramatically for SIs than for NPCIs.

Since neither SI nor NPCI methods were developed specifically for use with CIM, we developed and investigated a CIM-specific confidence interval estimation method, CIM-NPCI. Here we report an extensive simulation study that compares the two existing confidence interval estimation methods to our proposed method when using composite interval mapping at varying marker densities and at varying distances from the nearest genetic marker to the simulated QTL positions. We show that CIM-NPCIs consistently capture the simulated QTL across all sets of conditions, and that the slightly narrower SIs and NPCIs fail to do so at unacceptably high rates, especially when marker density is high. As high marker densities are essential in studies attempting to finely localize QTL, these findings are significant. Further, in examining the consequences of a known propensity for CIM LOD peaks to gravitate toward nearby genetic markers, we uncovered several trends that are instructive for considering how best to apply the CIM and CIM-NPCI methods to achieve optimal results.

## Methods

### Software

The QTL Cartographer v1.17 suite of applications [18] was used for all QTL simulations and QTL analyses. Python scripts (http://www.python.org) that perform the CIM-NPCI method were recently reported [20], but were revised extensively for this study in order to perform simulations of thousands of runs of thousands of resamplings. *R* scripts (http://www.r-project.org) were written and used to harvest and summarize the results from the analyses of simulated QTL data and are available upon request.

### Genetic Map Specification

The RIL population type (RI1) was selected for testing confidence interval methods because positional cloning efforts are currently best suited to high resolution plant populations of isogenic lines. A typical sample size of *n* = 200 lines was used. The linkage map was designed to have four 165 cM chromosomes with identical marker coverages. On each chromosome, 23 markers were placed such that their density varies progressively from high to low as cM distance from the short arm telomere increases (Figure 1). In order to specify this genetic map, a data file with the sequential intermarker distances was created and converted into map format (software specific) for simulating data. Specifically, using the QTL Cartographer software, an input file specifying this information was processed by Rmap in order to generate the RI1.map file used for simulating QTL effects in the next section [18].

### Simulation of QTL Effects

Only additive effects were simulated since we used an RI1 population design. For all simulations, heritability was held constant at 0.6, meaning that 60% of the total phenotypic variance could be explained by genetic effects. In all cases, four genetic effects were simulated with one on each chromosome to ensure genetic independence. So that effects of marker densities could be assessed and tracked, the simulated position of the QTLs within the 165 cM length of any of the chromosomes was deliberately varied from 1 cM to 20 cM intermarker distance (Figure 1). *Q_1_* was placed on chromosome 1 in the most marker-dense region, *Q_2_* on chromosome 2 in a moderately dense region, *Q_3_* on chromosome 3 in a moderately sparse region_,_ and *Q_4_* on chromosome 4 in the most sparse region of the generic chromosome (Figure 1). All four QTL were assigned equivalent additive effects, with an overall trait mean of zero, such that the scale of the phenotype data was irrelevant. On average then, each of the four QTL should have accounted for 15% of the phenotypic variance, since heritabilites of 0.6 were simulated.

In order to permit examination of the relationship between the simulated location of the QTL and the nearest genetic marker, four sub-simulations were conducted. In the first sub-simulation, the QTL were placed precisely at the left marker (0 cM distance). In the three other sub-simulations the QTL were placed at progressively increasing distances from the left marker, 10%, 30%, and 50% of the marker intervals where they were placed (Table 1). We designate the parameter D be the distance of the QTL from the left marker. For each sub-simulation, *N* = 1000 distinct RI1 sample data sets of size *n* = 200 were generated with the specified genetic map and QTLs. In the QTL Cartographer software, we used Rqtl to simulate datasets with QTL effects at assigned positions and equivalent effect magnitudes [18].

**Table 1:** QTL locations by flanking marker and sub-simulation.

					Distance from LM			
Chr.	QTL	LM	RM	Intermarker distance	0%	10%	30%	50%
1	*Q_1_*	11	12	1	11.0	11.1	11.3	11.5
2	*Q_2_*	23	26	3	23.0	23.3	23.9	24.5
3	*Q_3_*	69	84	15	69.0	70.5	73.5	76.5
4	*Q_4_*	114	134	20	114.0	116.0	120.0	124.0

*Notes*: One QTL (*Q_x_*) was placed on each of the four chromosomes (Chr.) in regions of varying marker density (from dense to sparse). For four subsimulations the QTLs were placed at varying distances from the left marker. The QTL locations and the left and right flanking marker (LM and RM) locations are indicated in cM.

### QTL Estimation and SI Extraction

CIM was performed on all 4,000 RI1 data sets. CIM is done by regressing the trait on each locus in the genome with some markers used as covariates and called background markers. Background markers for CIM were selected using stepwise regression of the trait on all markers with forward and backward elimination to determine which markers are significantly related to the trait (meet the p = 0.05 significance threshold). A hard bound of 10 was placed on the number of forward regression steps. In our simulations, this is done using SRmapqtl with model set to two (the model with forward and backward selection in stepwise regression) in the QTL Cartographer software [18]. CIM was implemented using up to five background markers as cofactors (covariates in the regression of trait on locus). The specific loci to be tested for significance with CIM are determined by the walking speed or distance between test sites. The walking speed was set such that test positions were spaced at 1.0 cM intervals throughout the genome. Since all marker positions were specified as whole numbers, exactly 166 positions were tested per chromosome per run. A 10 cM blockout window (any markers in that window are blocked) on each side of the test position was used to exclude nearby background markers from being covariates in the regression model. The use of a blockout window should avoid multicollinearity arising from linkage between loci and nearby markers. Specifically, using the QTL Cartographer software, the Zmapqtl function was used with the model set to six (the model with up to five background markers used as covariates and a 10 cM blockout window around the test position) [18].

LOD curves of all four QTL in each of the 4,000 RI1 data sets were constructed and used to extract 2.0 LOD SIs, which are more conservative than 1.5 LOD SIs. For all 16,000 simulated trait-locus associations, the width of each SI and whether or not it contained the assigned QTL position were recorded and summarized.

### The CIM-NPCI Method

The CIM analysis yields a LOD profile plot for the genome. These LOD scores are based on regressing a quantitative trait on each locus in the genome with some markers designated as background markers and used as covariates in the regression. When the locus is a strong predictor of the quantitative trait (while controlling for the background markers), the LOD score will be large. We expect that a significant peak in LOD score reveals a QTL at that approximate location.

There are many loci in the genome and we must be careful about choosing a threshold for significance to avoid making many Type I errors. A model-free method for setting the experiment-wise Type I error rate is using a permutation test. This is done by permuting the trait values among the individuals in the dataset *P* times and calculating the LOD scores using CIM for each permuted data set. LOD scores which are large compared to this set are considered significant and only a peak LOD score that exceeds this threshold is considered for calculating a CIM-NPCI.

To create a confidence interval for the location of the underlying QTL creating a significant LOD peak, we use bootstrapping techniques. One bootstrap sample consists of a resampling with replacement the same size as the original dataset. A bootstrap dataset may include one observation from the original data several times, but unlike a permutation dataset the trait values are always paired with the same genetic data as in the original dataset. A 95% interval calculated from bootstrapped datasets will have 95% coverage without making distributional assumptions.

To calculate a CIM-NPCI, *B* bootstrap datasets are generated and the peak of the LOD for each chromosome is calculated. Mimicking the “selective method” for NPCI estimation developed for Haley-Knott regression methods [14], [19], only LOD peaks which exceed the significance threshold for the original dataset are considered significant. When analyzing these bootstrap datasets, we perform complete CIM analysis on each one including determining appropriate background markers. So, the threshold for significance is set by the original data, but the background markers used in CIM are specific to each analysis of bootstrapped data.

The locations of the LOD peaks for bootstrap datasets which exceed the threshold are ordered. The central 95% of these location values defines the 95% CIM-NPCI for QTL location. An alternative method is to order all the LOD peak positions without applying the selective method and calculate the central 95%. We call these intervals non-selective CIM-NPCIs.

### Bootstrapping and CI Estimation

The same 4,000 RI1 data sets were resampled by bootstrapping. We implemented this bootstrapping in the QTL Cartographer software using Prune [18]. *B* = 1,000 bootstrap datasets were created from each of the 4,000 RI1 datasets by sampling with replacement until *n* = 200 individual observations were obtained. All 4,000,000 bootstrap data sets were analyzed by CIM as described above. The LOD curves were created for these 16,000,000 trait-locus associations and the peak heights and positions were recorded. The permutation significance threshold was calculated from *P* = 1,000 permuted datasets. We implemented this bootstrapping in the QTL Cartographer software using Prune [18]. From the peak heights and the significance threshold, 16,000 CIM-NPCIs and 16,000 non-selective CIM-NPCIs were calculated as previously described. The width of each CI and whether or not it contained the assigned QTL position were recorded and summarized.

Since it has been used in the literature on simulated and real CIM results [21], we also tested the bootstrap CI method implemented in QTL Cartographer, which lacks the background marker selection step after each new bootstrap data set is created [18]. We term this method NPCI estimation for its similarity to the Visscher *et al.* method [14], however statistically incorrect this application of NPCI may be. Using Python scripts, CIM was implemented on all 4,000 RI1 data sets with all of the same parameters as described above, except that for each RI1 data set, the original set of background markers selected during initial analysis of the simulated dataset was used in the analysis of all *B* = 1,000 resampled datasets. Just as for CIM-NPCI calculations, the 16,000,000 trait-locus associations were interrogated to produce 16,000 NPCIs and 16,000 non-selective NPCIs. The width of each CI and whether or not it contained the assigned QTL position were recorded and summarized.

### Statistical Comparison of Methods

The aim of this study is to compare several positional confidence measures for QTL detected by CIM. For assessing performance of these methods, both coverage probability and interval width are metrics of interest in the context of high resolution QTL mapping studies that aim to localize genetic effects so narrowly that positional cloning and demonstrating pleiotropic action are reasonable next steps. Coverage probabilities are obtained by calculating the percentage of simulations where the constructed confidence interval actually captures the true parameter, in this case, the QTL position. If a 95% confidence interval method is appropriate, then one would expect 95% of the simulations to result in confidence intervals that capture the location of the true QTL. Interval widths are equally important; a confidence measure that achieves adequate coverage via wide intervals that are too conservative is sub-optimal, although the consequences of conservatism are typically preferred over those of under-coverage in this context.

## Results

### Coverage Probabilities

**Figure 2:**
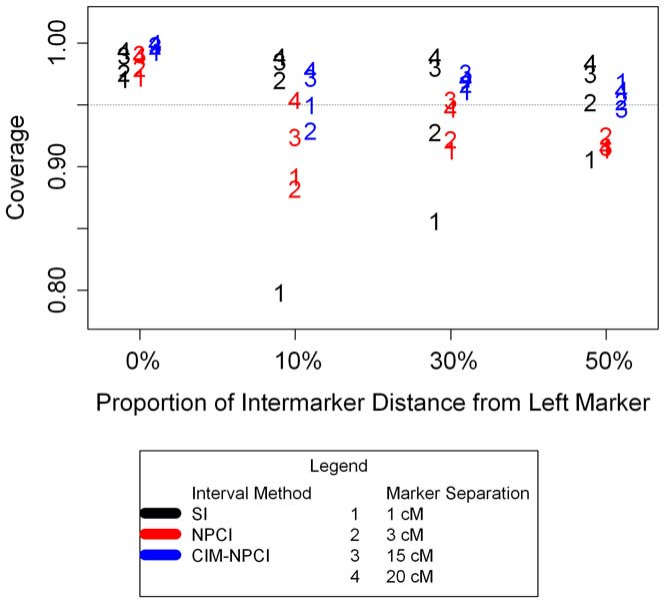
Plot of coverage probabilities of confidence interval methods. For the four QTL positions relative to the nearest marker (sub-simulations), interval coverage probabilities are shown for the four marker densities examined. SI (black), NPCI (red) and CIM-NPCI (blue) coverage probabilities are plotted for *Q*_1_, *Q*_2_, *Q*_3_ and *Q*_4_ (denoted by chromosome number only). The dashed line indicates the desired 95% coverage level.

Independent of genetic marker density, the SI, NPCI and CIM-NPCI methods are all too conservative when the QTL are placed precisely at markers; the first sub-simulation (0% distance) results show that all three coverage probabilities are >95% for *Q*_1_, *Q*_2_, *Q*_3_ and *Q*_4_ (Table 1; Figure 2). For the sub-simulation where the QTL are placed 10% of the inter-marker distance away from the left marker, the SI method performed very poorly for the QTL in a dense marker region, *Q*_1_, but was slightly conservative for the other QTLs, *Q*_2_, *Q*_3_ and *Q*_4_ (Figure 2). Performance of the SI method remains inadequate, but improves for *Q*_1_ across sub-simulations in sparse marker regions where the QTL are placed 30% and 50% of the inter-marker distance away from the left marker (Table 1, Figure 2). By contrast, performance of the SI method is worse for QTL in a moderately dense region, *Q*_2,_ in sub-simulations with QTLs far from markers (30 & 50% distance) compared to QTLs on and close to markers (0 & 10% distance), demonstrating that marker density and QTL placement each affect CIM accuracy differently.

NPCIs have sub-optimal coverage probabilities for all four loci in the sub-simulations with QTLs in the middle of two markers (50% distance), and for several of the loci in sub-simulations with QTLs that are 10 and 30% of the interval distance away from the flanking marker (Figure 2). For SIs and NPCIs, a pattern of decreasing spread among coverage probabilities for QTL in all marker densities is visible across sub-simulations with the QTL not on the left marker (10–50% distance). This pattern is only extensible to CIM-NPCI for the sub-simulation with QTLs at 10% distance from the left marker, where all three methods show their largest spreads in coverage probabilities for QTL capture across the four marker densities (Figure 2). CIM-NPCIs give insufficient coverage in only this one case, where *Q*_2_ falls below 0.95. Otherwise, CIM-NPCIs perform well, with *Q*_1_, *Q*_2_, *Q*_3_ and *Q*_4_ clustering more tightly in the coverage probability plots than they do for SIs and NPCIs, indicating that they are less variable as a function of the local marker density (Figure 2). On average, CIM-NPCIs were closest to the 95% coverage level that is desired.

### Interval Widths

Averaged across loci for all four sub-simulations, interval widths are relatively similar: 9.64, 10.79 and 11.34 cM for the SI, NPCI and CIM-NPCI methods, respectively (Table 2). Since the NPCI method shows the worst coverage probabilities and does not have the narrowest intervals (Figure 2; Table 2), further discussion of NPCI results is minimized. When SI and CIM-NPCI widths are compared, SIs are 17% narrower when averaged across all conditions. If only the cases where both of these methods had >0.95 coverage probabilities are considered, average widths are 11.47 and 13.04 cM, making SIs 14% narrower (Table 2). Averaged across the four cases where SIs are anti-conservative and CIM-NPCIs have appropriate coverage, 5.79 and 7.61 cM average widths are observed, making SIs 31% narrower than CIM-NPCIs (Table 2).

**Table 2:** Average interval widths by confidence interval method, marker density and sub-simulation.

Distance from LM	Method	*Q_1_* IW	*Q_2_* IW	*Q_3_* IW	*Q_4_* IW
0%	SI	4.8 (.19)	4.4 (.06)	9.3 (.08)	11.5 (.10)
	NPCI	4.5 (.27)	4.1 (.26)	5.4 (.27)	6.3 (.33)
	CIM-NPCI	7.5 (.13)	7.5 (.13)	8.9 (.14)	11.1 (.17)
10%	SI	*5.0 (.10)*	4.9 (.07)	11.6 (.12)	14.5 (.12)
	NPCI	*7.4 (.38)*	*7.8 (.39)*	*11.7 (.54)*	14.4 (.60)
	CIM-NPCI	7.1 (.11)	*7.5 (.12)*	10.7 (.16)	13.2 (.17)
30%	SI	*6.1 (.20)*	*6.0 (.08)*	13.9 (.12)	17.3 (.22)
	NPCI	*7.6 (.37)*	*8.2 (.39)*	16.5 (.72)	*21.5 (.92)*
	CIM-NPCI	7.5 (.13)	8.4 (.12)	16.1 (.21)	20.0 (.27)
50%	SI	*6.0 (.11)*	6.2 (.08)	14.7 (.21)	17.8 (.17)
	NPCI	*7.4 (.38)*	*8.4 (.39)*	*18.3 (.73)*	*22.3 (.90)*
	CIM-NPCI	7.4 (.11)	8.6 (.12)	18.1 (.22)	21.6 (.28)

*Notes*: For the four QTL positions (dense marker region to sparse marker region *Q_1_—Q_4_*) of each sub-simulation, interval widths (IW) are averages from *N* = 1,000 simulations and are given in cM with standard errors denoted in parentheses. An IW in italics indicates that its coverage probability is below 0.95.

### Selective versus Non-Selective CI Estimation

Similar to findings of Visscher and colleagues [14], selection of the bootstrap resamplings to include on the basis of whether or not the QTL peak exceeded the Type I error rate threshold had almost no effect on coverage probabilities or interval widths. For the comparison of CIM-NPCI to non-selective CIM-NPCI, pairwise correlation coefficients across the 16 sub-simulation by QTL combinations are *r* = 0.99 (p<0.0001) for coverage probability and *r* = 0.99 (p<0.0001) for interval widths. However, CIM-NPCIs were 0.18 cM narrower than non-selective CIM-NPCIs on average, a statistically significant (p<0.001), but negligible difference. The NPCI to non-selective NPCI comparisons follow these trends exactly (not shown). If a lower heritability or a smaller population size were used, one might expect to see a greater benefit from using the selective method.

### Marker Density versus Chromosome End Effects

Coverage probability results for *Q_1_* were different than those from the other three loci in several sub-simulations (Figure 2; Table 2). Since it is possible that the proximity of the *Q_1_* locus to the end of the chromosome could account for its different behavior, we performed additional simulations to test whether or not similar proximity to the chromosome end could affect coverage probabilities for a simulated QTL placed between widely spaced markers. Using an identical map and the same experimental framework, *Q_1_* was relocated to cM positions 154, 152, 148 and 144 in four respective sub-simulations of *N* = 1000 RI1 data sets. These positions represent 0%, 10%, 30% and 50% of the distance from position 154 toward position 134. These sub-simulations spatially mirror the original tests of *Q_1_* coverage probabilities because the “0%” sub-simulation places *Q_1_* 11 cM from the telomeric markers, which occur at cM positions 0 and 165. In the subsequent sub-simulations, *Q_1_* was simultaneously moved toward the midpoint between the two markers and away from the chromosome end (see Table 1 and Figure 1 for comparison). *Q_1_* coverage probabilities for both SIs and CIM-NPCIs in these four modified sub-simulations were all above 0.95 (data not shown), closely matching what was observed for *Q_4_*, which represented the sparse marker density condition in the primary study (Figure 2). These results suggest that marker density, rather than proximity to the end of the chromosome, explains the variation in *Q_1_* coverage probabilities observed in the primary study.

## Discussion

Improved genetic resolution and availability of sequenced genomes have made positional cloning of moderate-effect QTL realistic in several systems, emphasizing the need for precise and accurate derivation of positional confidence intervals (CIs). To investigate the performance of SI, NPCI and CIM-NPCI methods for estimating positional confidence for QTL detected by CIM, 4,000 RI1 data sets each simulating four equivalent and moderate QTL effects were generated and analyzed by all three methods. We found that one of the three methods, NPCI, performs very poorly and that neither SIs nor CIM-NPCIs are perfect, although they each have strengths for particular applications.

### Balancing Exactitude and Accuracy

If two confidence interval methods attain the same coverage probability, then the narrower of the two intervals would be considered better because it places narrower bounds on the value of the true parameter while maintaining the same level of confidence. However, interval widths are of no consequence when coverage probabilities are inadequate. CIM-NPCIs give consistently adequate, but slightly conservative coverage. As a result, they are 14% wider than SIs in the subset of cases where coverage probabilities for both methods are acceptable. While it is possible to calculate this percentage with the simulated data available, analysis of real data sets has no such luxury.

With a real QTL, one can not know whether the allelic difference underpinning the phenotypic effect resides at a marker, or 10% of the way toward the next marker. In these sub-simulations, CIM-NPCIs perform well, while SIs perform admirably for the first, but atrociously for the second. One must then conclude that SIs perform poorly when marker density is high (Figure 2). It is true that CIM-NPCIs are substantially wider for *Q_1_* and *Q_2_* across all four sub-simulations, but this should be viewed as an acceptable cost for accuracy and certainty. It is precisely because they are slightly wider that CIM-NPCIs encompass the QTL with appropriate coverage; the bias of CIM toward preferentially locating QTL effects at markers is overcome by the interrogation of 1,000 bootstrap samples of the dataset. While a single LOD curve can be artificially steep and lead to an SI that does not contain the true QTL location, it is much less likely that peak positions for resampled data sets will fall too close together and miss the true location.

In cases where marker density is as low as it is surrounding *Q_3_*, CIM-NPCI confers no advantage in coverage probability and incurs a moderate penalty for its wider interval widths (Tables 1 and 2; Figure 2). Thus, we do not recommend using the CIM-NPCI method for cases when marker density is low, and conclude that the SI method actually is appropriate for these cases.

### Application to Real Data

CIM-NPCIs were compared to SIs in two recent studies that used maize iRILs to obtain high genetic resolution [20; N. Lauter, W. Zhang, D. Hessel, S. Moose, in preparation]. The map for this population has an average intermarker distance of 3.2 cM, so many of the intervals are quite narrow. More than 40 QTL from six oligogenically inherited traits have been comparatively analyzed, and surprisingly, the CIM-NPCIs are ∼30% narrower than the SIs on average. It is possible that the levels of missing data or proportions of outlying datapoints in these studies are responsible for this difference from simulated results, which draw on complete data sets that collectively offer a full range of distributions. It is also possible that the 40 trait-locus associations examined are non-representative and that the observed difference in performance of these methods is artifactual. However, the CIM-NPCI did capture the QTL peak in every case, and in some cases, peaks for two traits are tightly overlapping to suggest pleiotropic action. In one case, the α = 0.05 CIM-NPCI is confined to a single BAC, confining the QTL to a region of about 160 kb (N. Lauter, W. Zhang, D. Hessel, S. Moose, in preparation). While this feat must clearly credit the iRIL strategy [3], the performance of the CIM-NPCI method on a dense genetic map with several markers per BAC in this region should not be overlooked. Application to additional real data sets will be required to further investigate whether or not CIM-NPCIs are consistently narrower than SIs in practice.

### Choosing a CI Method According to Purpose

Some good reasons to perform CIM-NPCI are that marker density is high and accuracy is critically important. For most other cases, it is not necessary to perform 1,000-fold more mapping runs than otherwise required. This decision is best made on a locus by locus basis, as marker density is not typically uniform on a map. That said, regardless of where a QTL is located relative to the flanking markers, the SI method seems appropriate to use on CIM results so long as intervals between markers remain greater than 3 cM.

The advent of new marker and breeding approaches will continue to change what we define as dense marker spacing. As transcript derived markers are increasingly used, maps will become more dense, and expectations of QTL cloning and establishment of pleiotropic action will go up. Collection of molecular phenotype data in large quantities is becoming more common [22], bringing increases in data volume that on the one hand are ill-suited to lengthy reinterrogation methods, but on the other hand are more likely to require them. As the phenotype of interest becomes more narrowly defined, its inheritance becomes increasingly tractable [1], [15]. Higher heritabilities lead to narrower LOD peaks, and when tens of thousands of trait-locus associations are all mapped, the challenge turns to testing for pleiotropic action, where confidence intervals become increasingly relevant [23], [24].

### Conclusions

We have observed that increasing the level of detail in a data set, which is often an interim goal for us as experimentalists, weakens the performance of the most widely used confidence measure, which had heretofore not been explicitly tested for its performance with CIM results. The plasticity of LOD curve shapes generated by CIM makes use of the SI method subject to bias, as the curve can be optimized for peakedness and then assessed for its spread. An attractive feature of an empirical computational method such as CIM-NPCI is that this bias is removed. Moreover, CIM-NPCI outperforms the SI method, which is surprising, because non-parametric bootstrapping is outperformed by parametric methods when applied to SIM results [25]. The discovery that CIM LOD curve shapes are especially sensitive to false-reporting (under-coverage) in marker-dense regions is significant, and the potential for the CIM-NPCI method to consistently provide narrower localizations with higher confidence is encouraging.
